# Cardiac dose in the treatment of synchronous bilateral breast cancer patients between three different radiotherapy techniques (VMAT, IMRT, and 3D CRT)

**DOI:** 10.1007/s12672-023-00636-z

**Published:** 2023-03-02

**Authors:** Nidal Salim, Alexey Popodko, Kristina Tumanova, Alexandr Stolbovoy, Irina Lagkueva, Vadim Ragimov

**Affiliations:** 1 Department of Radiation Oncology, European Medical Center, Moscow, Russia; 2 Department of Radiation Oncology, Russian Medical Academy of Continuous Medical Education of the Ministry of Health, Moscow, Russia; 3 Russian X-Radiology Research Center, Moscow, Russia

**Keywords:** Synchronous bilateral breast cancer, Radiation therapy, 3D CRT, IMRT, VMAT, Cardiac conduction system

## Abstract

**Purpose:**

Synchronous bilateral irradiation of both mammary glands and chest wall is a challenging task due to technical difficulties and limited evidence supporting an optimal technique to improve treatment outcomes. We studied and compared the dosimetry data of three radiotherapy techniques to select the most optimal one.

**Methods:**

We compared three-dimensional conformal radiation treatment (3D CRT), intensity-modulated radiation therapy (IMRT), and volumetric modulated arc therapy (VMAT) during irradiation of synchronous bilateral breast cancer in nine patients followed by examination of dose distribution to the cardiac conduction system (SA node, AV node and Bundle of His), myocardium, lungs, left anterior descending artery (LADA) and right coronary artery (RCA) .

**Results:**

VMAT is the most sparing technique for SBBC treatment. Even though doses to the SA node, AV node and Bundle of His were higher with VMAT (D_mean_ were 3.75 ± 0.62, 2.58 ± 0.83 and 3.03 ± 1.18 Gy respectively) compared with 3D CRT (D_mean_ were 2.61 ± 0.66, 1.52 ± 0.38 and 1.88 ± 0.70 Gy respectively), this difference is statistically insignificant. Doses to the right and left lung (average D_mean_ = 12.65 ± 3.20 Gy, V_20Gy_ = 24.12 ± 6.25%), myocardium (D_mean_ = 5.33 ± 1.51 Gy, V_10Gy_ = 9.80 ± 3.83%, V_20Gy_ = 7.19 ± 3.15%, V_25Gy_ = 6.20 ± 2.93%), and LADA (D_mean_ = 10.04 ± 4.92 Gy, V_20Gy_ = 18.17 ± 13.24% and V_25Gy_ = 15.41 ± 12.19%) were highest with 3D CRT. The highest  D_mean_ in the cardiac conduction system (5.30 ± 2.23, 3.15 ± 1.61 and 3.89 ± 1.85 Gy respectively) was observed with IMRT, and a similar effect was noted in RCA (D_mean_ = 7.48 ± 2.11 Gy).

**Conclusion:**

VMAT is the optimal and satisfactory radiation therapy technique for sparing organs at risk (OARs). With VMAT, a lower D_mean_ value was noted in the myocardium, LADA, and lungs. The use of 3D CRT significantly increases the dose of radiation reaching the lungs, myocardium, and LADA, which can subsequently cause cardiovascular and lung complications, but not in the cardiac conduction system.

## Introduction

Synchronous bilateral breast cancer (SBBC) is commonly referred to as two or more malignant neoplasms in the both mammary glands detected up to 6 months apart [1]. SBBC of the mammary glands accounts for 1.0–3.5% of all breast cancer cases [2]. SBBC staging is defined for each side separately as the treatment consists of the same steps as in unilateral breast cancer treatment. Depending on the disease stage (on each side), treatment approaches may include breast-conserving surgery (BCS)/mastectomy (with lymphadenectomy), neoadjuvant/adjuvant chemotherapy or hormone therapy, and radiation therapy. Administration of radiotherapy after BCS reduces the risk of local recurrence by 4–5% [3, 4].

In cases of bilateral breast cancer, dosimetric planning of radiation therapy is a particularly complex and time-consuming process. The use of traditional tangential fields is often associated with inhomogeneous dose distribution in the planning treatment volume (PTV) and with the hotspots formation [5–8]. The latter leads to complications such as radioepidermitis, telangiectasia, shrinkage of the mammary gland, and a subsequent decrease in the quality of life. However, the most serious danger is radiation damage to the myocardium and coronary vessels, as well as the occurrence of secondary malignant tumors, most often in the esophagus and contralateral breast in case of unilateral irradiation [9, 10]. Also, especially high doses on the heart and lungs were noted in some patients [6, 11–13].

According to the Early Breast Cancer Trialists Collaborative Group (EBCTCG) review in 2000, the mortality among radiation-exposed patients with secondary neoplasms is higher than that from primary breast cancer [9]. The 15-year accumulation rate of secondary malignant tumors is 16–19% in patients undergoing radiation therapy [10]. In 2013, Darby et al. investigated the consequences of radiation treatment of breast cancer in 2,168 women in Sweden and Denmark who were treated between 1958 and 2001. Of these, 963 were found to have cardiovascular issues, including myocardial infarction, ischemia with the need for myocardial revascularization, or death from myocardial ischemia [14]. The average dose to the whole heart was 6.6 Gray (Gy) for left-sided and 2.9 Gy for right-sided breast cancer. It was discovered that exceeding the average dose in these patients linearly increased the frequency of the above cardiac complications by 7.4% without a visible threshold. Nowadays, in the new radiation therapy era, it has become important to consider the low dose bath (LDB) effect. In fact, even 4% of a total 50 Gy prescribed dose to treat breast cancer which represent only 2 Gy may also affect the treatment safety.

The importance of studying the effect of radiation therapy on the cardiac conduction system and coronary arteries is clear from the side effects of radiation therapy for breast cancer in the cited literature. This is especially important when treating patients with heart disease. To the best of our knowledge, the significance of radiation damage to the cardiac conduction system in irradiation of SBBC has not been studied. We analyzed radiation exposure to the cardiac conduction system (sinoatrial (SA) node, atrioventricular (AV) node, bundle of His), coronary arteries (LADA, right coronary artery (RCA), myocardium and lungs. We also compared the dose distribution between the three main radiation therapy techniques: Three-dimensional conformal radiation treatment (3D CRT), intensity-modulated radiation therapy (IMRT), and volumetric modulated arc therapy (VMAT) in the studied organs at risk (OARs).

## Patients and methods

This was an open-label, comparative study focused on the treatment of SBBC, the radiation dose distribution over the PTV, and the dose distribution to OARs. Nine patients at the Radiation Oncology Department (European Medical Center, Moscow, Russia) from 2015 to 2019 participated in the study.

The main aim of our study was to determine the most optimal radiotherapy technique, considering dose to the cardiac conduction system in the treatment of patients with SBBC. This is an observational study. The Internal Ethics Committee has confirmed that no ethical approval is required.

All patients underwent standard examinations including diagnostic mammography, a biopsy and estrogen/progesterone receptor (ER/PR) and ERBB2 (HER2 or HER2/neu status) determination. Each patient underwent surgery and neoadjuvant chemotherapy/hormone therapy as recommended in the National Comprehensive Cancer Network (NCCN) guidelines. All patients had indications for adjuvant radiotherapy and were assessed by a multidisciplinary team comprising chemotherapy oncologists, oncological surgeons, and radiation therapists at the initial referral. Patient median age was 62 years (range 43–73). The detailed patient characteristics of disease stage and the scope of surgery shown in Table 1.

**Table 1:** Patient characteristics of disease stage and the scope of surgery

Patient characteristics				
№	Age (y.o.)	Staging (Right/Left breast)	Surgery (Right/Left breast)	PTV
1	56	T1N1cM0 T2N2bM0	Lumpectomy + ALND Lumpectomy + ALND	Mammary glands on both sides + I-III levels of axillary LNs, supraclavicular LNs, parasternal LNs on both sides
2	68	T1cN0M0 T1cN0M0	Lumpectomy + ALND Lumpectomy + ALND	Mammary glands on both sides + I-III levels of axillary LNs on both sides
3	64	T2N0M0 T1cN2aM0	Lumpectomy + ALND Lumpectomy + ALND	Mammary glands on both sides + I-II levels of axillary LNs on both sides
4	61	T1cN1aM0 T1bN0M0	Lumpectomy + ALND Lumpectomy + ALND	Mammary glands on both sides + I-II levels of axillary LNs on both sides
5	43	T2N1M0 T1N1M0	Mastectomy Mastectomy	Chest wall on both sides + I-III levels of axillary LNs on the left; I-III levels of axillary LNs, supraclavicular LNs on the right
6	73	T1cN1aM0 T1cN0M0	Mastectomy Mastectomy	Chest wall on both sides + I-III levels of axillary LNs, supraclavicular LNs, parasternal LNs on both sides
7	61	T3N2aM0 TisN0M0	Mastectomy Lumpectomy	Chest wall on the right + I-III levels of axillary LNs, supraclavicular LNs, parasternal LNs; mammary glands on the left
8	58	T1N0M0 T1N0M0	Lumpectomy + ALND Lumpectomy + ALND	Mammary glands on both sides
9	58	TisN0M0 T1cN0M0	Lumpectomy Lumpectomy + ALND	Mammary glands on both sides

^*^ALND axillary lymph node dissection, *LNs* lymph nodes

All patients underwent computed tomography (CT) on Brilliance CT Big Bore Philips (Philips, Amsterdam, Netherlands). CT-simulations were performed without contrast enhancement on the patient in the supine position with arms raised above the head at an angle of 120° to the median body axis using QUEST™ Breastboard RT-4543 (Qfix, Avondale, PA, USA). The breast board tilt was set to 5º for all patients. CT scans were performed with free breathing (FB) on the area between the lower jawbone and the diaphragm. Each patient was instructed to breathe smoothly and evenly during the CT simulation and subsequent treatment sessions. The chest movement was tracked during the CT simulation and the maximum difference in movement was 1.5—2 mm. We took into account this difference on PTV and used pseudo-skin flash method with virtual bolus. CT  slices  were 2 mm thick. Treatment plans were created using the computer planning system Eclipse (v. 15.6) by Varian Medical Systems (Palo Alto, USA). All patients received radiation treatment on Varian Truebeam linear accelerator with Millenium 120-multileaf collimator (MLC) manufactured by Varian Medical System (Palo Alto, USA). 3D CRT, IMRT, and VMAT plans were generated and compared dosimetrically using the same structure sets.

We investigated external irradiation with three radiation therapy techniques: 3D CRT, IMRT, and VMAT. We performed radiation therapy planning and subsequent radiation treatment with a VMAT treatment plan developed by a medical physicist and radiation oncologist. Additionally, for research tasks, 3D CRT and IMRT plans were generated in the treatment planning system. All plans were created using the same structure sets. Comparative assessment of the plans was made using dose-volume histograms (DVH) and cross-sectional analysis of dose distribution in PTV and OARs. A uniform basis for comparison was the use of the same contours for all three variants of the plan in one patient.

Treatment planning was performed according to the Radiation Therapy Oncology Group (RTOG) 1005 recommendations for outlining PTV with consideration of OARs. The criteria for adopting a PTV dose distribution plan were: V_95%_ (volume in % receiving 95% of prescribed dose) ≥ 95% of volume (≥ 47.5 Gy) and maximum dose (D_max_ = D_0.03 cc_ (dose to 0.03 cc volume) was less than 110%, D_max_ < 120% was considered acceptable in especially difficult cases. The Conformity index (CI) and the dose homogeneity index (HI) were calculated using the definitions below:

$${\text{CI }} = {\text{ BV}}_{{{95}\% }} /{\text{ PTV volume}}$$

$${\text{HI }} = {\text{ D}}_{{{5}\% }} /{\text{ D}}_{{{95}\% }}$$

where D_5%_ and D_95%_ equals the minimum dose to 5% and 95% of the PTV volume and BV_95%_ equals body volume of the 95% of prescribed isodose. The closer CI and HI are to 1, the better PTV coverage was.

The SA, AV nodes, and Bundle of His were not visible on CT scans; therefore, their locations were estimated based on human anatomy atlases. The studies of Saremi F et al., Malik S et al., P. Loap et al. and two studies by Stefenson R et al., were used to determine the components of the cardiac conduction system on computed tomograms [15–19]. We recorded the mean dose (D_mean_) and D_max_ for SA, AV nodes and Bundle of His.

For bilateral breast cancer radiation therapy treatment right and left lungs are usually considered separately [20]. We recorded separately for left and right lung D_mean_, V_20Gy_ (OAR volume in % receiving 20 Gy), V_10Gy_ and V_5Gy_, for myocardium—D_mean_, V_25Gy_, V_20Gy_ and V_10Gy_. When studying the distribution of LDB outside the treatment volume, we focused on their carcinogenic interval—from 2 to 10 Gy (4–20%) of the prescribed total dose. The indicated dose range was chosen based on previously reported ranges in studies of radiation-induced secondary malignant diseases arising after the treatment of the primary tumor [21–28].

Dual-isocentric approach was applied in 3D CRT and IMRT treatment planning. For 3D CRT plans each isocenter had two to four 6 MV and 10 MV photon beams. When PTV included upper-level lymph nodes field conjunction was performed. “Field-in-field” method and wedges were used in planning if necessary. For IMRT plans each isocenter had seven 6 MV or 10 MV photon beams equally distributed around tangential field angles (gantry angles between 179 and 300° for left and 181–60° for right isocenter respectively). 3D CRT plans were normalized such that 95% of the prescribed dose covered greater than 95% of the PTV, for IMRT if necessary, the normalization was applied such that PTV dose coverage was fulfilled.

**Fig. 1:**
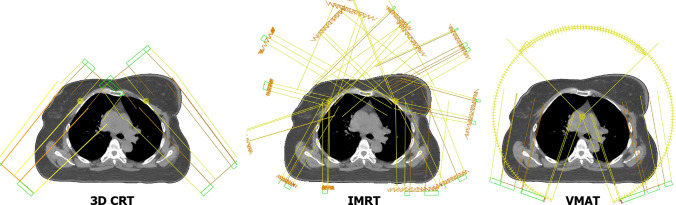
An example of fields positioning for 3D CRT, IMRT and VMAT treatment plans

The VMAT was performed by creating four arcs of gantry rotation with a single isocenter. Photon energies were also 6 MV. The Isocenter was located below the sternum by 4–5 cm. We selected the values of the angles of the beginning and end of the arc pairs ranging from 179° to 300° and 181° to 60° clockwise and counterclockwise. Collimator rotation for each arc was chosen individually and fitted to left or right breast depending on the arc pair. If necessary, the normalization was applied such that PTV dose coverage was fulfilled. Figure 1 shows an example of fields positioning for 3D CRT, IMRT and VMAT techniques.

We conducted data analysis using the non-parametric Kruskal–Wallis test for multiple samples, considering irradiation methods used (3D CRT, IMRT or VMAT). The Tukey’s honest significant difference (HSD) test was used for paired group comparison. All statistical tests were two-tailed. P < 0.05 denoted statistical significance.

## Results

Table 2 shows PTV coverage characteristics: the D_x%_ (dose (Gy) to x% of the PTV), V_y%_ (percentage of the PTV receiving  ≥ y% of prescribed dose), D_mean_, D_max_, CI and HI. VMAT technique showed the highest V_95%_ dose coverage volume in PTV with 98.71 ± 0.84% (p < 0.001). The largest high-dose volumes V_105%_ and V_110%_ was observed in 3D CRT—62.19% and 24.76% respectively followed by IMRT (31.52% and 3.98%) and VMAT (21.31% and 0.19%), p < 0.001. The D_max_ was lower for VMAT method than for IMRT and 3D CRT, which didn’t show a significant difference. The VMAT plans showed the best CI and HI values—1.02 ± 0.02 and 1.08 ± 0.01 respectively. IMRT gives better indicators of conformity (1.09 ± 0.03) and homogeneity (1.13 ± 0.02) than 3D CRT where CI was 1.43 ± 0.12 and HI—1.19 ± 0.02 (p < 0.001), since more beams are used, but this is accompanied increase in low dose volume, which is almost absent in 3D CRT.

**Table 2:** Comparison of PTV dose coverage characteristics using 3D CRT (1), IMRT (2), and VMAT (3) techniques

PTV Parameters	Treatment technique						p-value	p^(a)^		
	3D CRT^1^		IMRT^2^		VMAT^3^					
	Mean	SD	Mean	SD	Mean	SD		1 vs 2	1 vs 3	2 vs 3
V95% (%)	96.23	0.74	96.91	0.83	98.71	0.84	< 0.001	0.198	< 0.001	< 0.001
V105% (%)	62.19	5.63	31.52	9.08	21.31	11.56	< 0.001	< 0.001	< 0.001	0.063
V110% (%)	24.76	5.26	3.98	3.17	0.19	0.25	< 0.001	< 0.001	< 0.001	0.081
D98% (Gy)	43.78	2.81	46.77	0.53	48.03	0.69	< 0.001	0.003	< 0.001	0.272
D2% (Gy)	57.93	0.59	55.25	0.55	53.75	0.53	< 0.001	< 0.001	< 0.001	< 0.001
Dmean (Gy)	53.23	0.93	51.44	0.41	51.34	0.42	< 0.001	< 0.001	< 0.001	0.933
Dmax (Gy)	59.21	0.38	58.56	0.74	56.97	0.84	< 0.001	0.128	< 0.001	< 0.001
CI	1.43	0.12	1.09	0.03	1.02	0.02	< 0.001	< 0.001	< 0.001	0.195
HI	1.19	0.02	1.13	0.02	1.08	0.01	< 0.001	< 0.001	< 0.001	< 0.001

p^(a)^—post-hoc analysis using Tukey’s HSD test

Regardless of the side we observed similar findings in both lungs where VMAT showed better results than 3D CRT and alike with IMRT technique. The average mean dose (D_mean_) of left and right lung for VMAT was 7.72 ± 1.29 Gy, while for 3D CRT it was 12.65 ± 3.20 Gy and for IMRT—9.17 ± 2.12 Gy. Table 3 shows the statistical comparison of the OAR dose distribution for each treatment method.

**Table 3:** Comparison of the dose absorbed by at-risk organs under irradiation using 3D CRT (1), IMRT (2), and VMAT (3) techniques

OAR parameters	Treatment technique						p-value	p^(a)^		
	3D CRT^1^		IMRT^2^		VMAT^3^					
	Mean	SD	Mean	SD	Mean	SD		1 vs 2	1 vs 3	2 vs 3
Lung Left										
V20Gy (%)	23.77	5.20	12.88	4.78	7.34	4.07	< 0.001	< 0.001	< 0.001	0.050
V10Gy (%)	30.76	6.92	27.51	8.08	18.98	6.26	0.008	0.605	0.005	0.045
V5Gy (%)	41.42	10.41	47.56	8.52	46.72	9.40	0.251	0.370	0.472	0.981
Dmean (Gy)	12.36	2.82	8.72	1.85	7.36	1.40	< 0.001	0.003	< 0.001	0.372
Lung Right										
V20Gy (%)	24.47	7.31	15.33	6.16	9.78	2.96	< 0.001	0.007	< 0.001	0.124
V10Gy (%)	31.62	8.29	31.36	8.53	22.38	4.64	0.010	0.997	0.035	0.042
V5Gy (%)	43.13	9.13	48.71	9.80	48.27	8.65	0.341	0.417	0.475	0.994
Dmean (Gy)	12.93	3.57	9.63	2.39	8.09	1.19	0.003	0.031	0.002	0.425
Myocardium										
V25Gy (%)	6.20	2.93	0.36	0.99	0.01	0.03	< 0.001	< 0.001	< 0.001	0.912
V20Gy (%)	7.19	3.15	0.78	1.35	0.17	0.31	< 0.001	< 0.001	< 0.001	0.793
V10Gy (%)	9.80	3.83	10.27	4.98	3.39	4.92	0.012	0.975	0.018	0.011
Dmean (Gy)	5.33	1.51	5.41	1.18	3.99	0.75	0.054	0.988	0.064	0.047
LADA										
V25Gy (%)	15.41	12.19	0.13	0.36	0.50	1.50	< 0.001	< 0.001	< 0.001	0.993
V20Gy (%)	18.17	13.24	1.02	2.03	1.04	2.85	< 0.001	< 0.001	< 0.001	1.000
Dmean (Gy)	10.04	4.92	7.65	2.09	5.79	1.89	0.098	0.287	0.028	0.459
Dmax (Gy)	40.56	13.29	18.20	7.71	14.59	10.77	0.003	< 0.001	< 0.001	0.762
RCA										
Dmean (Gy)	2.91	0.68	7.48	2.11	4.76	1.52	< 0.001	< 0.001	0.046	0.003
Dmax (Gy)	5.03	2.33	13.76	5.11	8.35	3.22	< 0.001	< 0.001	0.164	0.014
His bundle										
Dmean (Gy)	1.88	0.70	3.89	1.85	3.03	1.18	0.006	0.010	0.180	0.376
Dmax (Gy)	2.32	1.06	4.74	2.07	3.07	1.44	0.009	0.009	0.565	0.085
SA Node										
Dmean (Gy)	2.61	0.66	5.30	2.23	3.75	0.62	0.003	0.001	0.219	0.068
Dmax (Gy)	3.50	1.04	7.08	4.05	4.51	0.90	0.021	0.014	0.665	0.090
AV Node										
Dmean (Gy)	1.52	0.38	3.15	1.61	2.58	0.83	0.001	0.010	0.110	0.508
Dmax (Gy)	1.63	0.42	3.39	1.70	2.68	0.91	0.001	0.008	0.140	0.404

p^(a)^—post-hoc analysis using Tukey’s HSD test

**Fig. 2:**
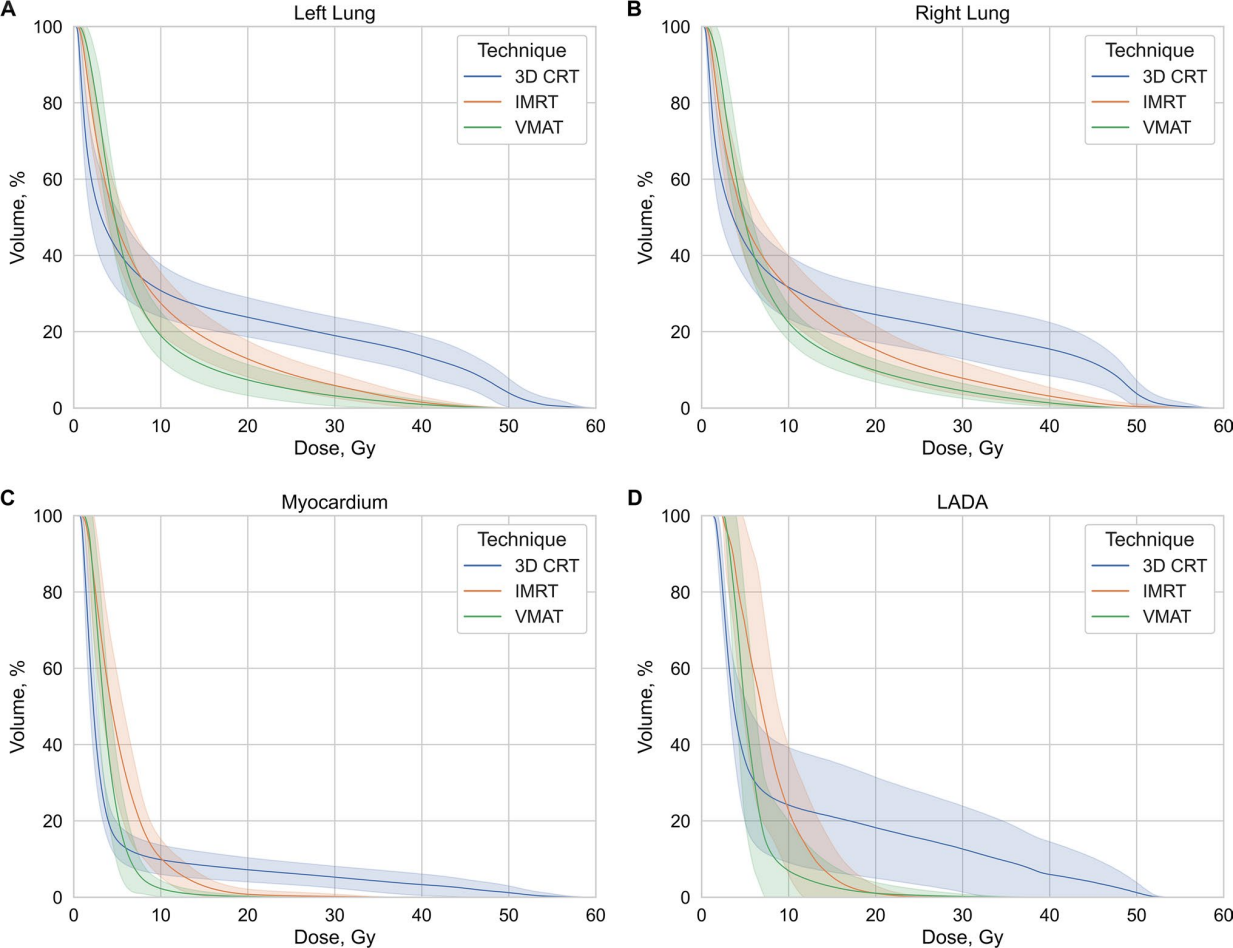
Comparison of DVHs between three radiation therapy techniques for left lung (**A**), for right lung (**B**), for myocardium (**C**) and for LADA (**D**). The blue line represents 3D CRT method, the red line—IMRT, the green line—VMAT. Colored shadows designate SD

The difference in V_5Gy_ was statistically insignificant between the methods with average value 45.97 ± 9.32%. As shown in Fig. 2A and B, significant variations appeared below and above of this 5 Gy point due to the techniques specifics. In dose range lower than 5 Gy, 3D CRT had better dose distribution than IMRT and VMAT and on the contrary for higher dose range. The average V_20Gy_ volume for both lungs for VMAT was 8.56 ± 3.51%, for IMRT was 14.11 ± 5.47% and for 3D CRT—24.12 ± 6.25% (p < 0.001).

The D_mean_ in myocardium among techniques was lowest with VMAT (3.99 ± 0.75 Gy, p = 0.054). Similarly, to lung, the LDB volume was lowest for 3D CRT, while high doses were lower for VMAT and IMRT as shown in Fig. 2C. The average V_20Gy_ volume in VMAT plans was 0.17 ± 0.31% and IMRT—0.78 ± 1.35%, while in 3D CRT it was 7.19 ± 3.15%. The only significant difference between VMAT and IMRT techniques in myocardium was observed in V_10Gy_ resulting in 3.39 ± 4.92% and 10.27 ± 4.98% respectively (p = 0.011).

For RCA 3D CRT plans showed lower D_mean_ values than VMAT and IMRT (2.91 ± 0.68 Gy vs 4.76 ± 1.52 Gy and 7.48 ± 2.11 Gy respectively). The D_max_ with 3D CRT was also lower than VMAT value (5.03 ± 2.33 Gy vs 8.35 ± 3.22 Gy, p = 0.014), while it was similar with IMRT.

Regarding the LADA D_max_, V_20Gy_ and V_25Gy_ showed insignificant differences between IMRT and VMAT plans and were significantly higher for 3D CRT (16.4 Gy, 1.03 Gy and 0.32 Gy against 40.56 Gy, 18.17 Gy and 15.41 Gy respectively, p < 0.001). In D_mean_ only significant difference was observed between 3D CRT and VMAT methods, where it was approximately 4 Gy lower for VMAT. The DVH difference between three techniques is shown in Fig. 2D.

**Fig. 3:**
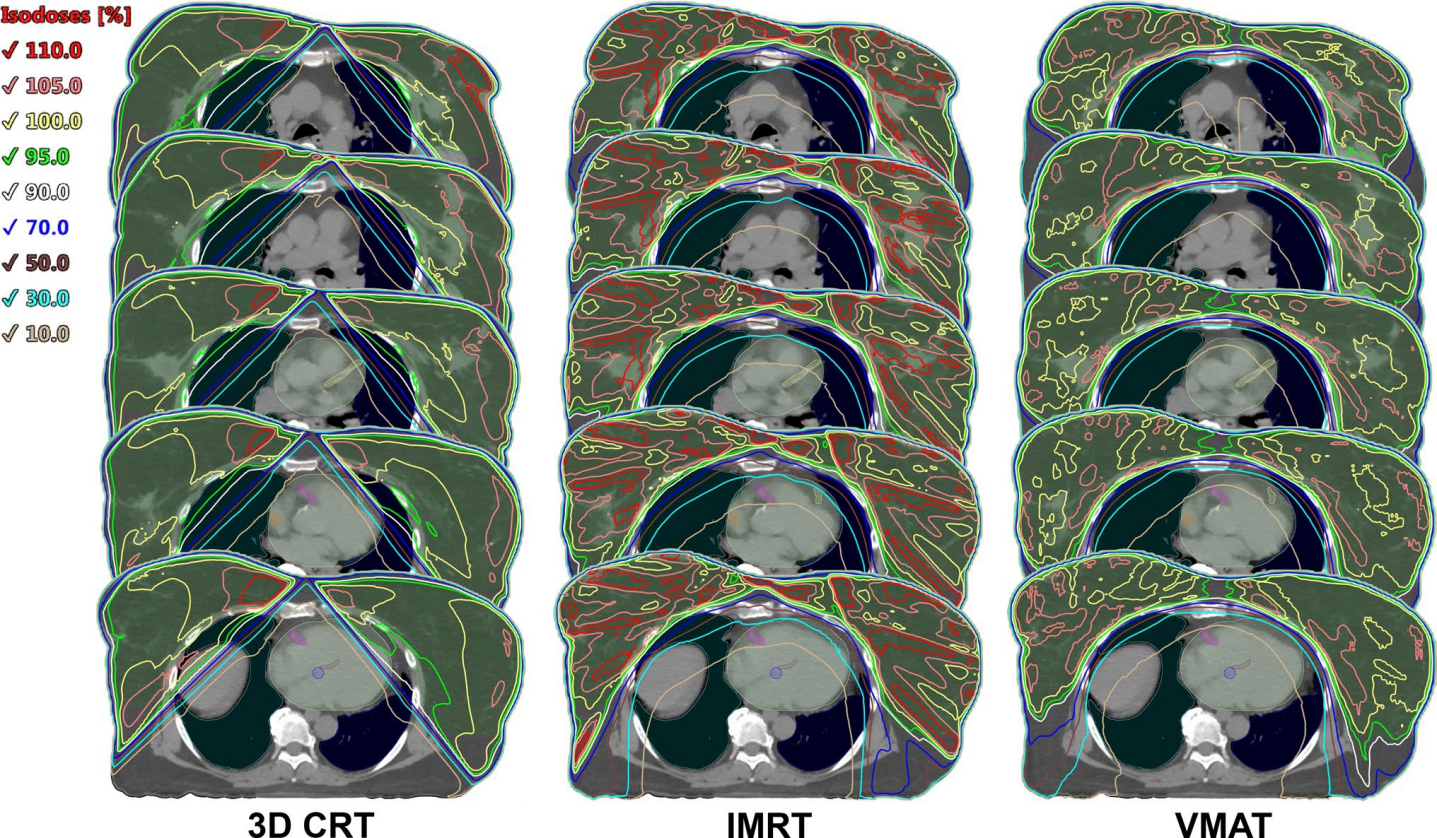
The dose distribution examples of SBBC treatment plans using 3D CRT (on the left), IMRT (in the middle) and VMAT (on the right)

Figure 3 shows the dosimetry variations of SBBC treatment plans for different techniques.

In SA node, AV node and His bundle the D_mean_ and D_max_ were lower for 3D CRT plans than for IMRT, while VMAT plans took intermediate values. In pairwise comparison statistically significant difference was observed only between 3D CRT and IMRT (p < 0.05). As it is shown in Table 3 the D_max_ for these three OARs with 3D CRT and VMAT was lower than 5 Gy and the differences between the methods were equal or less than 1 Gy.

**Fig. 4:**
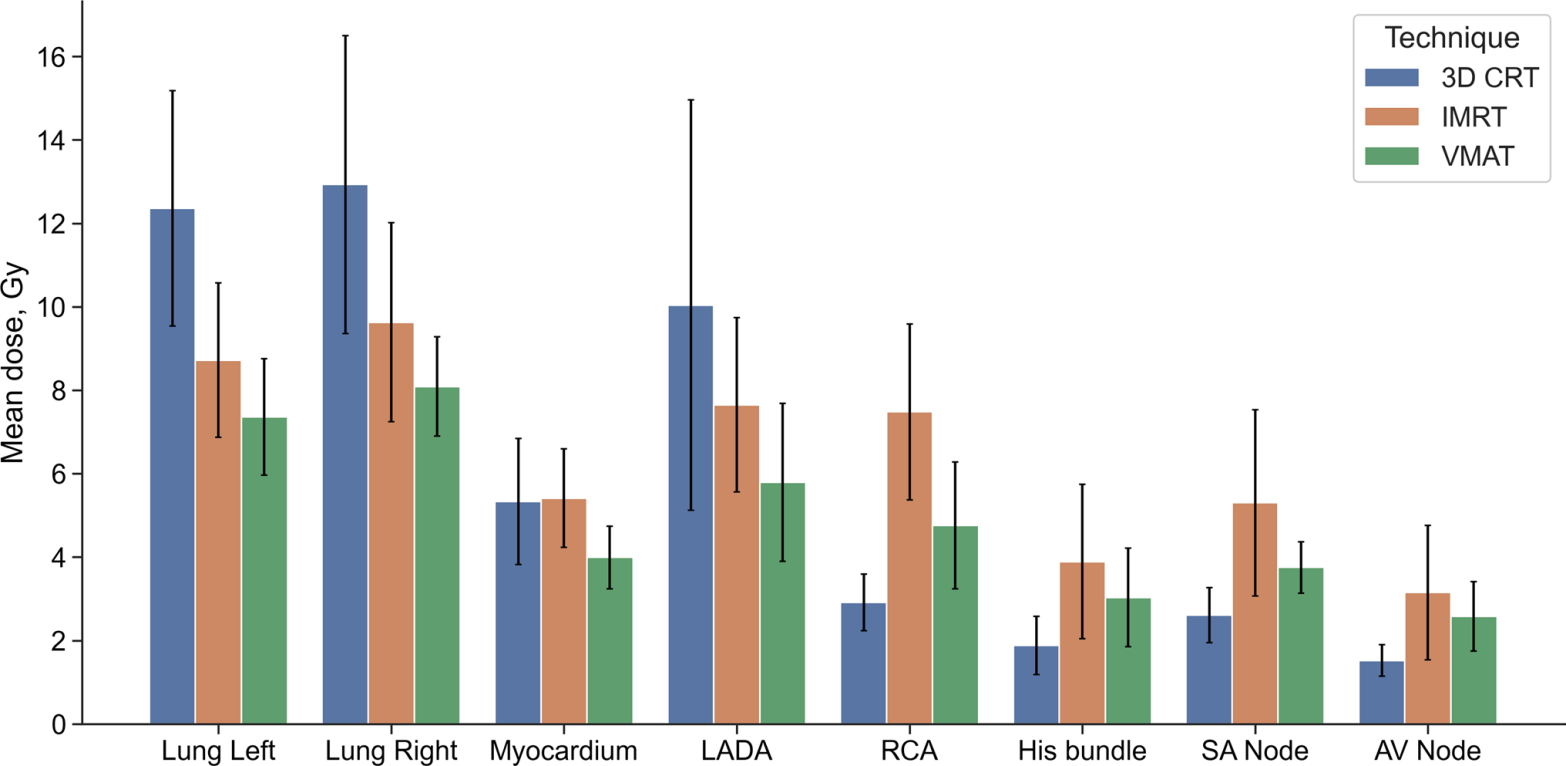
Mean dose with SD in the OARs of 3D CRT, IMRT and VMAT technique

The D_mean_ in the OARs (left and right lungs, myocardium, LADA, RCA, Bundle of His, SA and AV nodes) of 3D CRT, IMRT and VMAT technique are represented in Fig. 4.

## Discussion

The increase of cardiac complications begins within the first 5 years after radiation therapy treatment. However, the greatest number of complications was observed in patients with risk factors. 54% of patients with post-radiation coronary complications died from these causes. In fact, the frequency of events does not depend on dose to the LADA, but the dose to the whole heart. Based on these findings, radiation injuries may appear on the main coronary arteries and myocardial structures—cardiomyocytes and the microvasculature—are jointly involved in the pathogenesis of the most severe outcomes.

Wu et al. demonstrate that in the modern approach of combined treatment modalities, the ischemic attacks of the heart are replaced by arrhythmias and disturbances of intracardiac impulse conduction. Modern post-radiation arrhythmias were discovered to be as life-threatening as post-radiation myocardial infarction [29]. Several interesting articles have investigated the effect of radiation doses on the cardiac conduction system. P. Loap et al., evaluated exposure to the cardiac conduction system during breast irradiation with VMAT and estimated the potential dosimetric benefit with intensity modulated proton therapy (IMPT). The authors concluded that AV and SA nodes can be substantially exposed, especially for right-sided breast irradiation, while IMPT delivers virtually no dose to the SA or the AV nodes [30]. Then, in another study, the authors evaluated the radiation exposure of cardiac conduction system in patients treated for mediastinal Hodgkin lymphoma (HL) with deep-inspiration breath-hold (DIBH)-VMAT and analyzed the potential dosimetric benefit of IMPT over VMAT [31]. They observed that the SA node was substantially exposed during mediastinal HL radiotherapy with DIBH-VMAT; it could be hypothesized that this observation might be correlated with the high frequency of observed subclinical and clinical conduction and rhythmic disorders after mediastinal HL irradiation and may justify considering cardiac conduction system as potential OARs. In cases of breast cancer treatment, especially in the left side, DIBH may reduce the dose to the heart and LADA by increasing the distance between the heart and the treated breast [32, 33]. Tamilarasu S at el. studied the impact of DIBH techniques for early stage of SBBC and concluded that the use of DIBH significantly reduces the radiation doses to OARs [34]. In another study, S. Gaal et al. concluded in their article that DIBH is an excellent heart sparing technique in breast RT, but about one-third of the patients do not benefit from that otherwise laborious procedure or benefit less than from an alternative method [35] S. Tanguturi also analyzed the effect of DIBH on cardiac dose and came to conclusion that the improvement with DIBH was not uniform, patients should be scanned with both FB and DIBH. A beneficial effect was favored by younger age, greater body mass index (BMI) and larger inspirational lung volume changes [36]. We agree that DIBH should be used in the treatment of SBBC, however, in the case of our patients, the use of DIBH was not possible due to the long treatment time and age of the patients. If the DIBH would have been used, it might have reduced the cardiac doses and affected the comparisons. Contrariwise, there will be always patients to which DIBH could not be applied and for these reasons this comparative study is seen beneficial.

VMAT can be the most appropriate method in many treatment situations, but cannot be applied universally, and each case must be considered individually in order to get the best outcome. If we evaluate the effect of LDB, then VMAT has the largest impact. It is also higher in IMRT than in 3DCRT and, according to study by Hall et al., is accompanied by almost twice the frequency of secondary radiation-induced cancers in successfully treated patients [37].

An overwhelming majority of scientists agree that radiation treatment of left-sided breast cancer is associated with a higher risk of heart complications than treatment of right-sided tumors. Rehammar et al., reported that this association is especially true if we focus on ischemic complications or all cardiac complications [33]; however, this difference is practically nullified if arrhythmias and conduction disturbances can be distinguished from each other.

Although there is an opinion that the IMRT and VMAT methods are preferable in cases of SBBC, it is still unclear if their contribute to achievement of the optimal dose distribution into the PTV and to reduction of the total dose in OARs compared with 3DCRT [38–43].

## Summary

VMAT is the most sparing technique for SBBC treatment. Even though doses to the SA node, AV node and Bundle of His were higher with VMAT compared with 3D CRT, this difference are statistically insignificant. Doses to the myocardium, both lungs and LADA were highest with 3D CRT. The highest mean dose (D_mean_) in the cardiac conduction system was observed with IMRT, and a similar effect was noted in RCA. Optimal PTV dose coverage can only be achieved at the cost of high doses in the OARs. When irradiating the lungs, both VMAT and IMRT fared better than 3D CRT especially at doses higher than 10 Gy. Similar results apply to the dose in the myocardium.

In conclusion, the choice of irradiation method in SBBC is not obvious and depends on many individual factors: the stage on each side, size and shape of general (bilateral) PTV. Despite difficulties in imaging visualization, the absorbed dose in the volume at risk of the cardiac conduction system should be assessed. These parameters are decisive and will determine the choice between 3D CRT, IMRT and VMAT. Considering that the occurrence of SBBC is an uncommon event, we were able to enroll 9 patients in a single center. Further clinical observation based on large groups of patients is needed to confirm our findings.

## Data Availability

Available upon request.

## Abbreviations

3D CRT: Three-dimensional conformal radiation treatment
IMRT: Intensity-modulated radiation therapy
LADA: Left anterior descending artery
RCA: Right coronary artery
VMAT: Volumetric modulated arc therapy
